# Impact of Depression and Inflammation on the Progression of HIV Disease

**DOI:** 10.4172/2155-9899.1000423

**Published:** 2016-06-03

**Authors:** Yainyrette Rivera-Rivera, Fabián J. Vázquez-Santiago, Elinette Albino, María del C. Sánchez, Vanessa Rivera-Amill

**Affiliations:** Department of Basic Sciences, Ponce Health Sciences University/Ponce Research Institute, Ponce, PR 00716, USA

**Keywords:** HIV, Depression, Chronic inflammation, ART, Cytokines

## Abstract

The human immunodeficiency virus type 1 (HIV-1) epidemic has negatively affected over 40 million people worldwide. Antiretroviral therapy (ART) has improved life expectancy and changed the outcome of HIV-1 infection, making it a chronic and manageable disease. However, AIDS and non-AIDS comorbid illnesses persist during the course of infection despite the use of ART. In addition, the development of neuropsychiatric comorbidities (including depression) by HIV-infected subjects significantly affects quality of life, medication adherence, and disease prognosis. The factors associated with depression during HIV-1 infection include altered immune response, the release of pro-inflammatory cytokines, and monoamine imbalance. Elevated plasma pro-inflammatory cytokine levels contribute to the development of depression and depressive-like behaviors in HIV^+^ subjects. In addition, comorbid depression influences the decline rates of CD4^+^ cell counts and increases plasma viral load. Depression can manifest in some subjects despite their adherence to ART. In addition, psychosocial factors related to stigma (negative attitudes, moral issues, and abuse of HIV^+^ subjects) are also associated with depression. Both neurobiological and psychosocial factors are important considerations for the effective clinical management of HIV and the prevention of HIV disease progression.

## Introduction

The human immunodeficiency virus type 1 (HIV-1) remains one of the most important epidemics in the world today. Fortunately, the development and introduction of antiretroviral therapy (ART) regimens have improved life expectancy for HIV-infected individuals [1,2]. The HIV-infected population constitutes a heterogeneous group carrying a variety of comorbidities [2]. Although ART has dramatically improved the quality of life and clinical outcomes of HIV^+^ subjects [3–5], a significant fraction of HIV^+^ subjects remain burdened by non–AIDS-related comorbidities [2].

Approximately one third of HIV^+^ subjects seek clinical care at least once during the appearance of acute symptoms prior to the time of diagnosis [6]. Early HIV diagnosis is often difficult because of the onset of non-specific and general symptoms [6,7]. Within the acute stages of infection, symptoms include general manifestations such as fever, malaise, anorexia, sore throat, arthralgia, headaches, and lymphadenopathy [8]. The development of gastrointestinal symptoms is also common among HIV^+^ subjects and these include: ulcers, nausea, diarrhea, abdominal pain, and weight loss [8]. However, the most common comorbid ailments include cardiovascular disease [9], bone fractures [10], non–AIDS-associated cancers [11], kidney disease [12], and neurocognitive impairments [13]. HIV subjects may also develop psychiatric conditions, such as anxiety disorders, manic symptoms, atypical psychosis, or depression (or any combination of two or more of the previous) [14]. However, neuropsychiatric comorbidities are usually underdiagnosed or overlooked when patients seek primary care, even though depression affects overall disease progression in the HIV-infected population.

In this review, we discuss the literature pertaining to the effects of HIV-related psychiatric comorbidities, particularly depression, over the adherence to ART regimen, inflammation, prognostic outcomes, and subsequent HIV-disease progression. In addition, we review psychosocial and neurobiological factors that influence the manifestation of depression and depressive-like behaviors in this population.

### Antiretroviral therapy (ART): HIV as a chronic disease

HIV-1 infection has shifted from being a deadly disease to being a chronic and manageable one. The early use of ART prevented approximately 13,500 new infections during 1996 to 2009 [15]. Patients infected with HIV are living longer [1,2] but have a higher prevalence of both AIDS-related [16] and non–AIDS-related comorbidities and risk factors [9–11]. Indirect evidence of such is seen in the decreased mortality rates among the infected population and the reduced number of HIV-related hospitalizations [17,18]. During the pre-ART era, the median survival of an AIDS patient ranged from five to twelve months after the confirmed diagnosis of one or more opportunistic infections, such as cytomegalovirus [19]. The advent of ART brought a dramatic reduction in AIDS-defining illnesses and mortality [20], benefiting HIV-infected individuals by ameliorating the rapid rate of CD4^+^ T cell decline [21] and by altering molecular viral dynamics [22]. The adherent use of ART for about a year successfully suppresses HIV-1 plasma RNA to less than 50 copies, allowing patients to strengthen their defenses [23]. The literature describing the advantages of ART regimens is reviewed elsewhere [24].

However, ART does not cure HIV-1, and, once it is initiated, it must be used throughout the life of the patient and with almost perfect treatment adherence [25]. Estimates indicate that patients require a treatment adherence of 95% in order to achieve optimal viral suppression [26]. Thus, after CD4^+^ cell count declines, antiretroviral non-adherence is the strongest predictor for progression to AIDS and, subsequently, death [27–29]. This shows that mortality risks and life expectancies in infected subjects are directly related to adherence to therapy [29]. Unfortunately, although there is a significant decrease in AIDS-related mortality [16], HIV-1–infected subjects will most likely continue to confront non-AIDS comorbidities, the presence of which can ultimately lead to poor quality of life and disease progression.

In developed countries, where ART is available and accessible, the majority of HIV-infected patients die from causes other than AIDS [2]. Not surprisingly, the HIV-infected population develops a higher frequency of organ-specific diseases than the HIV-negative population [30]. Indeed, HIV-related mortality and its complications have decreased due to the implementation of ART regimens [17,18]. Despite the current success in treating HIV infection, an outpatient study indicates that the prevalence of non-AIDS mortality increased from 13.1% to 42.5% since 1996 through 2004 [17]. In this study, patients who died from non–AIDS-related diseases had been initiated on ART at higher CD4^+^ counts and were receiving ART for longer periods than were the matched subjects who developed AIDS [17]. Lifson and colleagues determined the frequency of underlying causes of death among infected subjects from three randomized HIV trials during 1999 to 2008 [31]. They reported that the frequency of AIDS-defining illnesses accounted for 10% of mortality whereas non–AIDS-defining malignancies accounted for 21% of mortality. The Charlson comorbidity index (CCI) is a method of categorizing the comorbidities of patients. Based on CCI scores, the aging HIV^+^ population has a greater rate of comorbid burden than members of the general population do [30]. This evidence strongly suggests that subjects who are HIV-infected and undergoing ART will most likely develop at least one non–AIDS-defining morbidity during the course of disease. In addition, results on population-based analyses of the US registry centers showed that aging represents an important risk factor for the development of certain malignancies, such as rectal and lung cancers [32], and cumulative evidence indicates that members of the HIV-infected population are more susceptible to an accelerated aging process than their uninfected counterparts [33]. However, the underlying mechanisms remain elusive. It is still unclear whether the HIV-infection alone, the comorbidities that develop, or additional risk factors are responsible for the accelerated aging process in HIV-infected subjects [33].

### Comorbid depression in the HIV-infected population

Depression is a multifactorial disorder with signs and symptoms that combine to influence the affective, behavioral, cognitive, and somatic areas [34]. According to the Diagnostic and Statistical Manual of Mental Disorders, Fifth Edition (DSM-V), major depressive disorder (MDD), also known as clinical or unipolar depression, is a disorder characterized by the presence of a depressed mood for at least two weeks. Depression comprises an array of disturbances that negatively affect sleep, weight, appetite, pleasure- and reward-seeking behaviors and loss of motivation, among others [35,36]. In fact, depression is the primary cause of disability in the population aged from 14 to 44 years [37]. According to Murray et al., depression is projected to become the second leading cause of disability by the year 2020 [38]. Even in cases in which depression is treated efficiently, more than 75% of those depressed patients will experience recurrent episodes, and around 10 to 30% will experience residual symptoms [39,40]. Individuals who are depressed can lose an estimated 5.6 hours of productivity per week [37] and have a higher probability of being unemployed [41]. In terms of costs, depression accounts for more than $43 billion in medical care services and around $17 billion in productivity loss each year [42].

Several studies have reported variability in the prevalence of depression among the HIV^+^ population. Initial studies indicated that the prevalence of neuropsychiatric abnormalities was about 41% among HIV-infected patients in Zaire [43]. In an attempt to estimate the global prevalence of mental comorbidities, depression was assessed in a cohort from the WHO Neuropsychiatric AIDS study. The population included a representative sample of five major geographic areas affected by the HIV-1 epidemic [44]. Results from the mean global scores of the Montgomery–Asberg Depression Rating Scale showed that depression is higher in symptomatic seropositive individuals than in matched sero-negative controls [44]. A subsequent study using a nationally representative sample of US HIV^+^ subjects from the HIV Cost and Services Utilization Study (HCSUS) reported that major depression and dysthymia were respectively present in 36% and 26.5% of HIV cases [45]. The authors concluded that psychiatric comorbidity rates are at least four times higher in the HIV^+^ than in the general US population [45]. Using the Profile of Mood States (POMS) Depression-Dejection scale (55% sensitivity and 84% specificity) as a screening tool of depressive-like comorbidities among HIV-infected subjects, Patterson et al. identified 48% of HIV-infected patients as having MDD comorbidity [46]. Later, using a cross-sectional design, Silveira et al. showed that in a Brazilian cohort of HIV-infected individuals on ART, 32% of the participants presented depressive symptoms [47]. Using the Beck Depression Inventory (BDI) to evaluate the presence and intensity of depressive symptoms, the study identified most of the participants with depression as having mild (14%) to moderate (14%) symptoms. Only 4% presented severe manifestations of depressive symptoms [47]. Together these observations strongly suggest that depressive comorbidities are highly likely to develop and subsequently manifest in HIV-diagnosed subjects, regardless of where they live.

Within the HIV population, depression is the second most common psychiatric disorder, following substance abuse [48]. It is frequently underdiagnosed and goes untreated for long periods [49,50]. Findings by Leserman and colleagues indicate that the proportion of individuals suffering from both HIV and comorbid depression has reached 50% [51]. Even more, the NeuroAIDS Tissue Consortium study reported that 60% of the cases studied had lifetime episodes of major depression [52]. Estimates from the CHARTER cohort study indicate that episodes of major depression during a lifetime were present in 47 to 57% of cases with neurocognitive impairment [13]. Comorbid MDD accelerates HIV disease progression, jeopardizes adherence to antiretroviral treatment, and is a potential risk factor for viral transmission [14]. Depression may also negatively affect quality of life due to punishment behaviors, lack of social support, and poor self-esteem [53]. Furthermore, these variables may compromise adaptive coping styles which consequently alter adherence to ART [53]. According to a cohort study in Bostwana (n=300), in which HIV^+^ patients were on antiretroviral regimens, depression was considered a statistically significant predictor of non-adherence to ART within 6 months of initiation [54]. Do et al. also described that 28.3% of ART respondent (n=85) exhibited some level of depression (46.9% mild, 31.8% moderate and 21.1% severe) based on a modified battery of neuropsychiatric tests including the European Quality of Life (EQ-5D) instrument, and the BDI [54]. However, the authors state that one limitation of their study included the inability to assess long-term outcome in medication adherence and depressive states [54]. Therefore, when conducting regular screening of HIV-infected patients for comorbidities, clinicians must be certain to take note of any signs of depression or any depressive-like symptoms.

In addition to depression, ART-associated toxicity remains an important barrier to treatment adherence in HIV^+^ subjects. The modification of antiretroviral treatment, defined as toxicity-driven substitution of at least one antiretroviral, was evaluated in 1,558 patients from a HIV/AIDS cohort study from Brazil [55]. The ART modification incidence due to toxicity within the first year of ART initiation was considerable, 14.6%, and higher in individuals who were ≥ 40 years old [55]. Therapy modification due to toxicity in 3% of the total number of study subjects resulted from CNS manifestations including depression, insomnia, and phobia among others [55]. The mechanisms whereby ART may trigger CNS manifestations remain unclear. However, neuropsychiatric symptoms such as anxiety, depression, and attention disturbances may be related to the type of antiretroviral treatment used. Neuropsychiatric manifestations have been linked to the use of efavirenz (EFV) in some HIV^+^ subjects [56]. High levels of EFV in plasma (>2.74 μg/mL) were associated with suicidal ideation and depressive symptoms. Patients with EFV regimes that contained doses greater than 2.74 μg/mL were 5.68 times more likely to develop toxicity within the central nervous system [56]. In 2012, Silveira et al. found no statistical differences between the use of EFV and the development of depressive symptoms in HIV patients; however, they reported a 28% reduction in relative risk for depressive symptoms among subjects not receiving EFV [47]. Pedrol et al. studied the neuropsychiatric outcome in 129 HIV^+^ patients (38.7% with depression) who received Nevirapine as a substitute to EFV (89.9%) [57]. Nevirapine yielded a significant improvement in neuropsychiatric symptomatology including depression, sleep disturbances, anxiety, and attention deficits by 3 months of medication switch [57]. Future studies are warranted to further clarify the association of depressive-like symptoms with specific antiretroviral treatments to establish the early intervention of comorbid depression at the initiation of ART.

In terms of HIV disease progression, having depression can significantly increase plasma viral load—even after effective ART is initiated [58]—and accelerate the decline of CD4^+^ cell counts [58]. HIV-1 mainly infects CD4^+^ lymphocytes and monocyte-derived macrophages and consequently leads to the onset of AIDS and AIDS-related symptoms [59–62]. A well-known feature of early clinical impact on the immune system is a decline in the T cell CD4^+^/CD8^+^ ratio, resulting in a quick impairment of the cellular immune response [8]. The CD4^+^/CD8^+^ ratio decline has been reported to occur in 92% of sero-converters, whereas it occurs in only 40% of sero-negative individuals [8]. In addition, depression may also double the rate of CD4^+^ decline and negatively affects CD4^+^ counts at baseline [58]. Hence, depressive behavior, feelings of hopelessness, and low education levels at baseline may all—together or individually—be predictive in terms of causing the worsening of CD4^+^ and viral load levels [58]. In addition, depression in HIV^+^ individuals negatively affects the rate of cognitive symptoms [14]. In fact, depression is associated with higher deficits in the cognitive domains of language, comprehension, attention, and memory, among others [14,63]. Alterations to cognitive status may foster failure to adhere to ART, particularly in memory-impaired HIV^+^ subjects. Meta-analytic data showed that depressed patients are three times more likely than their non-depressed counterparts to be non-adherent to medical treatment [64]. Lastly, there is evidence that depressive symptoms in individuals during the course of HIV are predictive of worsened survival rates [65].

### Stigma, social rejection and low income

Psychosocial factors contribute to the variability in the clinical outcomes of HIV disease [58]. One key issue of HIV stigma is that infection is often associated with behaviors that are condemned by society, promoting prejudice, discrimination and abuse against patients [66,67]. Stigma represents an epidemiological barrier since a proportion of HIV-infected patients do not—and will not—get tested due to the perceived fear of rejection [68,69] and abuse [67]. Thus, adverse effects on the mental health of HIV^+^ subjects may result from such stigma generating feelings of anxiety and depression [68]. The depression that often develops in HIV-infected patients is thought to be attributable to the stress that attends being diagnosed with a highly stigmatized chronic condition [68,70]. The social stigma may cause some HIV^+^ individuals to refuse to disclose their HIV/AIDS diagnosis [67,69]. Furthermore, HIV-related stigma is an important stressor that leads to poor ART adherence [70]. In some cases, HIV-infected individuals tend to isolate themselves to prevent stigma-related negative attitudes [69]. A broad analysis of the physiological and mental effects of stigma revealed that stigma accounts for problems with adherence to ART, missed clinical visits, and depressive symptoms [70]. Low-income is also a socio-demographic factor significantly associated with the appearance of depressive symptoms (p=0.02) [56].

### Inflammation: cytokines and chemokines modulating the immune response

Biological factors appear to play key roles as well. During the initial acute stage of HIV infection, a potent immune response is observed in HIV-infected individuals compared to matched individuals infected with either hepatitis virus B or C [71]. Interleukins such as IL-1, IL-2, IL-6, IL-10, IL-15, IL-18 and cytokines such as IFN-α, IFN-γ, and TNF-α are elevated in the plasma of recently HIV-infected subjects [71]. Stacy et al. studied how these dynamics show up during HIV, HBV and HCV infections, and described two time-points — early and delayed — where the levels of certain cytokines increase [71]. For a high proportion of HIV^+^ participants, the pro-inflammatory factors IFN-α, IL-15, and TNF-α, among others, showed early and quick kinetics as viremia increased [71]. This was followed by an anti-inflammatory response in some of the participants. In contrast to the acute phase cytokine/chemokine response in HIV^+^, participants infected with HCV or HBV infections, did not exhibit a rapid exponential increase in the plasma levels of cytokines as viremia increased [71]. Correlation of these patterns of immune modulators expression with depression assessment establishes the association of depression symptoms with the expression of inflammatory mediators in plasma [72]. In addition, autoimmune conditions, such as rheumatoid arthritis (RA), are characterized by persistent inflammation and continued activation of both innate and adaptive immune responses [73]. In RA, the constant release of chemokine and cytokine mediators lead to the chronic inflammation of the synovium, inducing cartilage and bone destruction [74]. Elevated pro-inflammatory cytokines have been found in RA patients with anxiety and depression symptoms [75] and pharmacological agents which specifically inhibit inflammatory mediators provide evidence of reduction in depression and anxiety symptoms [76].

For HIV^+^ individuals, chronic inflammation may represent an etiological factor for the development of depression. The rise in cytokine levels induces sickness behavior, with symptoms such as malaise, lethargy, anxiety, and depression [77–79]. Using a Luminex-based assay to characterize a broad panel of plasma cytokines, our group provided further evidence of the correlation of elevated pro-inflammatory cytokines in the plasma of HIV^+^ individuals with depression symptoms [80]. In all likelihood, the induction of systemic pro-inflammatory responses may increase susceptibility to brain pathology. Inflammatory cytokines, namely IL-1β, IL-6, and TNF-α, are elevated in the plasma and brains of depressed patients [81]. In the brain, HIV-1 proteins activate perivascular macrophages and microglia triggering a cascade of pro-inflammatory cytokine expression [82]. These studies highlight systemic inflammation as an important correlate for the development of depression during the course of HIV infection [80,83–85]. Even though ART alleviates to some degree the viral-induced neurotoxicity within the brain [86], inflammation negatively affects disease outcome at advanced stages of HIV-infection [87]. Thus, the regulation of immune responses plays a pivotal role in the pathophysiology of comorbidities linked to HIV-associated depression [88,89].

### Neurobiological factors contributing to depression

Another effect of pro-inflammatory cytokines is the activation of the hypothalamic–pituitary–adrenal (HPA) axis, which is the regulator of the stress response [88,90]. When the stress axis is not properly regulated, the activation of HPA pathways can modulate monoamine expression in the CNS, consequently leading to symptoms of depression [91]. It is well known that the neurochemical imbalance of monoamines, such as serotonin (5-HT), norepinephrine (NE), and dopamine (DA), is linked to the development of depression. Studies in HIV-infected patients demonstrate impaired levels of dopamine among distinct brain regions [92,93]. The basal ganglia appear to be the most vulnerable among cognitive-impaired subjects [92]. In addition to alterations in the basal ganglia, evidence points to dopaminergic deficits in other brain regions. In fact, HIV-infected subjects demonstrate greater levels of impaired fronto-striatal functioning than their HIV-negative matched controls do [93]. Impairments in fronto-striatal functions were associated with dopamine imbalance within this cohort [93]. Alterations to the dopamine system are evident in post-mortem brain tissues from HIV-infected subjects, compared to those of uninfected controls [92]. Interestingly, a deficiency in subcortical dopamine was also present in the ART-adherent HIV-infected group [92]. It appears that the dopaminergic deficiency that leads to symptoms of depression in HIV-infected subjects may result from the complex impairment of distinct brain regions. The HPA axis also is affected by activated pro-inflammatory cytokines.

### Animal and human studies for HIV and depression

Several studies in animal models demonstrate a link between HIV viral factors and the appearance of depressive symptoms. Although HIV-1 does not infect mice or rats, there are murine and rat models designed to address the role of inflammation in the CNS caused by viral proteins such as gp120 [94,95] and Tat [82]. Inflammatory responses localized within the CNS may lead to the development of behavioral changes, including the manifestation of depression. In a rat model of viral neuropathology, the intracerebral injection of recombinant HIV-1 gp120 IIIB provoked neuroinflammatory responses that led to changes in behavior [94]. Specifically, the intraventricular injection of 0.5 to 2.0 μg of recombinant HIV-1 gp120 protein induced depressive-like behavior and brain gene expression of IL-1β and TNF-α [94]. In particular, weight loss, decreased food consumption, and reduced locomotor activity were observed among gp120-treated rats that did not receive anti-inflammatory treatment. The same research group determined that the activation of the HPA axis was in parallel to the manifestation of depressive-like and sickness behavior after 24 hours of intracerebral administration of HIV-1 gp120 [95]. In this model of gp120-induced inflammation and depression, animals demonstrated increased levels of prostaglandins (PGE_2_) *ex vivo* compared to saline controls [95]. The induction of PGE_2_ exhibited dose-dependent increments: 0.5 μg and 2 μg HIV of gp120 IIIB, respectively. After three hours of gp120 injection, the responses driving gp120-induced PGE_2_ production were significantly ablated by the preadministration of indomethacin (4 mg/kg), a non-steroidal anti-inflammatory drug, and were further evident after eight hours of treatment [95].

Similar to gp120, the viral protein Tat induces neuroinflammatory responses that are associated with depressive and sickness behaviors in an HIV neurotoxicity mouse model [82]. The intraventricular injection of 40 ng of Tat promoted not only sickness or depressive behavior but also up regulated the inflammatory gene expression of IL-1β, TNF-α, and IL-6 [82]. Mice treated with HIV-1 Tat were more prone to remain immobile during a forced swim test (as a measure of learned hopelessness) and exhibited decreased sucrose consumption compared to control mice [82]. Thus, in mice showing depressive-like symptoms, the gene expression of indolamine oxidase (IDO), the tryptophan-degrading enzyme, was elevated by four hours of a single dose of HIV Tat. The mechanism whereby Tat mediates overexpression of IDO remains elusive but mechanistic insights have been previously addressed [96,97]. Interestingly, induction of systemic inflammation by LPS in BALB/c mice increases IDO mRNA and activity by 24 hours of treatment [98]. Up-regulation of IDO mRNA and activity among aged mice (20–24 months old) significantly differed between saline- and LPS-treated groups. A significant turnover of both tryptophan and serotonin metabolisms ensued with increased plasma IL-6 and brain IDO levels [98]. On the other hand, fractalkine-deficient mice, that had been injected with lipopolysaccharide (0.5 mg/kg), showed persistent microglial activation preceding the presence of depression [99]. Such an observation highlights the ability of both HIV gp120 and Tat to induce inflammatory-mediated comorbid depression in an HIV-relevant setting of neuropathology. One limitation of these animal models is the fact that it does not account for the role of HIV replication within CNS, as HIV does not infect mice or rats. In addition, the cellular source of inflammatory-induced stimuli by HIV Tat is not described [96]. The cellular source of IDO expression and neuroinflammation point to astrocytes and microglia [82,99]. Summed together, these studies strongly suggest that inflammation is important for the development of depressive-like behavior in mice and that proinflammatory cytokines can play a pathophysiological role in the aging brain. Furthermore, in the absence of CNS viral replication, HIV-relevant inflammatory cytokines such as IL-6 may play a pivotal role in etiology of comorbid depression as a result of the presence of viral antigens such as HIV gp120 and HIV Tat.

In contrast, the daily administration of the cyclooxygenase type 2 inhibitor (COX-2), meloxicam (1 mg/kg), reduced the hippocampal inflammatory gene expression profile without improving depressive-like behavior in HIV-1 transgenic rats [100]. In their study, Nemeth and colleagues evaluated adolescent female HIV-1 transgenic rats [100], which may account for the differences seen when compared to rat models of intracerebral administration [82,94,95]. Albeit these studies are based on distinct experimental designs, the overall results may succinctly suggest that there is a gender-specific difference in the manifestation of inflammation-dependent depression in the presence of viral proteins. Therefore, pharmacological approaches should consider gender-specific rationales for the effective treatment of viral-induced CNS inflammation. A cross-sectional epidemiological study that screened depression in Western European and Canadian HIV-positive subjects reported that the prevalence of depressive symptoms was greater in women than it was in men (17.9% vs. 14.3%; *p* <0.01). The prevalence of depression for women and for patients undergoing ART were 20.8% and 10.6%, respectively. [83]. No statistical significance was observed between men and women undergoing ART (p value=0.71), indicating that antiretroviral therapy is crucial to preventing the development of depressive comorbidities [83]. Although this study did not evaluate immune or inflammatory markers [83], the suppressive effects of ART on neuroinflammation are well known [86]. Of interest, our group found elevated levels of the pro-inflammatory cytokines interleukin 15 (IL-15), interferon-gamma inducible protein 10, and granulocyte colony–stimulating factor [80]. It seems likely, then, that the attenuation of pro-inflammatory cytokines will result in the amelioration of depressive manifestations during HIV-relevant systemic inflammation. However, since the activation of the inflammatory cytokine cascade is complex, additional caution must be in place for assessing the side effects of therapies targeting inflammatory cytokines in patients with HIV.

The plasma profile of monoamine metabolites were evaluated in a study assessing depression comorbidities in HIV-seropositive and - seronegative patients from three independent cohorts [101]. Using Bioplex methods to determine plasma cytokine profiles HIV-seropositive and 36-HIV-seronegative patients, Cassol et al. found that their results distinguished depressed subjects from their non-depressed HIV-positive and HIV-negative counterparts [101]. In particular, diminished levels of tryptophan derivatives and metabolites correlated with the severity of depressive symptomatology [101]. In this study, the authors concluded that holistic approaches targeting inflammatory responses and mitochondrial-associated monoamine catabolism may be important in preventive treatments against comorbid depression during the course of HIV-infection.

## Conclusions

The HIV epidemic has caused millions of deaths in over 30 years since its discovery. While early initiation of ART has been shown to improve health, suppress viral load, and reduce transmission [102], there are still several clinical challenges that need to be addressed. One particular problem in the 21^st^ century is that the HIV-infected population has to deal with non-AIDS illnesses, including organ-specific diseases and neuropsychiatric comorbidities. Additionally, the relationship between the manifestation of depression and its impact on clinical markers such as CD4^+^ cell counts and HIV plasma viral loads remains elusive. Interestingly, the similarity of some symptoms shared by both conditions hinders the accuracy of diagnosis for depression in primary health care. Henceforth, a better understanding is required to effectively treat comorbid depression during the course of HIV disease. We also highlighted the importance of neuropsychiatric treatment directed towards depressive symptoms in HIV^+^ individuals as an adjuvant approach to the standard use of ART. However, additional research studies are required to elucidate the specific underlying mechanisms for developing depression among HIV infected subjects.

As mentioned in this review, depression is highly prevalent within the HIV population. The most common psychosocial and physiological factors that influence the development of depression in HIV^+^ population are HIV-related social stigma, the neurochemical imbalance of monoamines, and uncontrolled inflammation. Clinical practitioners should consider holistic approaches to ameliorate the development of non-AIDS comorbidities and age-related comorbidities. For example, clinicians should focus on ART adherence. Do and colleagues provided evidence that patients should be monitored in their first 6 months of treatment and 1 year after initiation of ART since some patients tend to discontinue their treatment because they feel better [54]. In addition, they mentioned the importance of incentives or motivational testimonies from other people living with HIV/AIDS in order to maintain those patients focused on the importance of taking ART [54]. Another study with 19 HIV patients from a public hospital in KwaZulu-Natal, South Africa, pointed out the importance of managing disabilities, including mental health issues in HIV patients [103]. They concluded that HIV-related disabilities need to be managed by rehabilitation professionals within patients’ homes and communities to be more effective [103]. Thus, professionals in primary health care settings who work directly with the HIV-infected population have to promote the engagement and retention of HIV-infected patients on these care services. The Centers for Disease Control and Prevention (CDC) has issued the “Recommendations for HIV Prevention with Adults and Adolescents with HIV in the United States” were they provide the guidelines on biomedical, behavioral and structural interventions aimed at reducing the infectiousness of HIV^+^ individuals and reducing the risk of exposing others to HIV [104]. The CDC report also highlights the incorporation of medical and social support services for individuals with HIV to help improve health outcomes [104].
